# Characterization of imaging performance of a novel helical kVCT for use in image‐guided and adaptive radiotherapy

**DOI:** 10.1002/acm2.13648

**Published:** 2022-05-15

**Authors:** Riley C. Tegtmeier, William S. Ferris, John E. Bayouth, Jessica R. Miller, Wesley S. Culberson

**Affiliations:** ^1^ Department of Medical Physics School of Medicine and Public Health University of Wisconsin‐Madison Madison Wisconsin USA; ^2^ Department of Human Oncology School of Medicine and Public Health University of Wisconsin‐Madison Madison Wisconsin USA

**Keywords:** adaptive radiotherapy, ClearRT, computed tomography, helical tomotherapy, IGRT, image quality, Radixact

## Abstract

ClearRT helical kVCT imaging for the Radixact helical tomotherapy system recently received FDA approval and is available for clinical use. The system is intended to enhance image fidelity in radiation therapy treatment planning and delivery compared to the prior MV‐based onboard imaging approach. The purpose of this work was to characterize the imaging performance of this system and compare this performance with that of clinical systems used in image‐guided and/or adaptive radiotherapy (ART) or computed tomography (CT) simulation, including Radixact MVCT, TomoTherapy MVCT, Varian TrueBeam kV OBI CBCT, and the Siemens SOMATOM Definition Edge kVCT. A CT image quality phantom was scanned across clinically relevant acquisition modes for each system to evaluate image quality metrics, including noise, uniformity, contrast, spatial resolution, and CT number linearity. Similar noise levels were observed for ClearRT and Siemens Edge, whereas noise for the other systems was ∼1.5–5 times higher. Uniformity was best for Siemens Edge, whereas most scans for ClearRT exhibited a slight “cupping” or “capping” artifact. The ClearRT and Siemens Edge performed best for contrast metrics, which included low‐contrast visibility and contrast‐to‐noise ratio evaluations. Spatial resolution was best for TrueBeam and Siemens Edge, whereas the three kVCT systems exhibited similar CT number linearity. Overall, these results provide an initial indication that ClearRT image quality is adequate for image guidance in radiotherapy and sufficient for delineating anatomic structures, thus enabling its use for ART. ClearRT also showed significant improvement over MVCT, which was previously the only onboard imaging modality available on Radixact. Although the acquisition of these scans does come at the cost of additional patient dose, reported CTDI values indicate a similar or generally reduced machine output for ClearRT compared to the other systems while maintaining comparable or improved image quality overall.

## 1 INTRODUCTION

The focus of modern‐day radiotherapy on highly specialized treatments to increase the precision and visualization of target volumes has led to the development and improvement of image‐guided radiotherapy (IGRT) in which patients are imaged before or during treatment delivery. Pretreatment images are registered with reference images obtained during the treatment planning process to improve tumor localization and patient positioning, thus minimizing the effects of intrafractional motion on geometric accuracy. A key advantage of IGRT is the ability to spare critical organs at risk (OARs) while allowing for highly conformal treatments and dose escalation, leading to reduced toxicity and improved tumor control.^1^ Good image quality influences the accuracy of the image registration process, avoiding issues associated with low‐quality images such as erroneous patient setup and treatment delivery.^2^ It is therefore necessary that the performance of these imaging systems be of sufficient quality to ensure safe and effective IGRT implementation.

However, in many instances simply repositioning the patient is not sufficient to fully correct for certain anatomical changes over the course of treatment delivery, such as variations in the target volume and OARs due to patient weight loss or tumor shrinkage. Adaptive radiotherapy (ART), defined as changing the original radiation treatment plan during a course of fractionated radiotherapy to account for the temporal changes in anatomy or changes in tumor biology or function, has progressed significantly since Yan et al introduced this concept over two decades ago.^3^, ^4^ Modifications to the original treatment plan in response to these changes include refining patient contours and target volumes and/or adapting the dose prescription or treatment plan altogether to be more or less aggressive based on additional learned information.^5^ However, much like with IGRT, the ability to perform accurate contour refinement and dose reconstruction for ART depends on the imaging system's ability to sufficiently delineate anatomic structures.

Computed tomography (CT) is the most popular methodology to obtain volumetric information required for IGRT and ART. ClearRT, a helical kVCT system, has recently been developed by Accuray Incorporated (Sunnyvale, CA) and is available for use on applicable versions of the Radixact helical tomotherapy system. This imaging solution is designed for integration with applications such as Synchrony real‐time motion synchronization and adaptive treatment delivery technology on the Radixact system.^6^ ClearRT recently received 510(k) clearance from the U.S. Food and Drug Administration and is available for consumer use.

At the time of this study, there exist no known publications that analyze the image quality characteristics of ClearRT to evaluate its fidelity for use in IGRT and ART. As a result, the goal of this study was to evaluate the imaging performance of the new system under possible clinical settings in comparison with other common CT imaging modalities utilized in IGRT and/or dose calculation: TomoTherapy MVCT, Radixact MVCT, Varian TrueBeam OBI kV CBCT (Varian Medical Systems, Palo Alto, CA), and Siemens SOMATOM Definition Edge CT Scanner (Siemens AG, Munich, Germany).

## 2 METHODS

### 2.1 Imaging systems evaluated

The ClearRT system consists of a kV X‐ray source and a flat‐panel detector (FPD) mounted orthogonal to the MV beam on the Radixact system. During treatment, the patient is continuously translated through the bore, whereas the beam rotates, forming a helical pattern. Image acquisition on the ClearRT system occurs in an identical manner, providing continuous helical imaging for up to 135 cm with a maximum scan rate of 1.7 cm/s and a maximum gantry rotation rate of 10 RPM.^6^ Clinically, the system can be utilized for both IGRT and ART image acquisitions by making use of an analytical reconstruction algorithm of filtered backprojection (FBP) with a Hilbert transform filter.^7^ Although the user cannot directly adjust the tube technique (kVp and mAs), the selection of specific protocols (named according to their expected use) specifies scan acquisition parameters. These parameters are summarized in Tables 1 and 2. The selection of a specific anatomy determines the kV energy and reconstructed slice interval, whereas the choice of body size configures the fluence of the beam to account for an appropriate imaging dose based on patient size. The field of view (FOV) determines filtration type and whether the detector and transverse collimation are offset from their centered positions (to attain a larger FOV). The field size is set by two *x*‐axes and two *y*‐axes 2‐mm‐thick tungsten blades automatically positioned based on the preset scan protocol. The mode selection defines the longitudinal beam width, couch speed, and views per rotation that impact the scatter signal, scan time, and patient dose. Note that based on longitudinal beam widths given in Table 2, ClearRT provides a unique interface between fan‐beam CT and cone‐beam CT (CBCT) geometries. For additional information on protocol specifications and available parameter combinations, as well as image dose information, the reader is referred to the Radixact Physics Essentials Guide.^7^

**TABLE 1:** Scan acquisition parameters for ClearRT determined by the selection of anatomy and body size

Anatomy	Body size	mA per view	kV	Slice interval (mm)	Slice thickness (mm)
Head	Small	80	100	1.2	2.4
	Medium	125			
	Large	160			
Thorax	Small	80	120	1.8	3.6
	Medium	125			
	Large	160			
	X‐large	200			
Pelvis	Small	80	140	1.8	3.6
	Medium	125			
	Large	160			
	X‐large	200			

*Note*: Anatomy determines the nominal beam energy and reconstructed slice interval, whereas anatomy and body size determine the mA per view.

*Source*: From Radixact Physics Essential Guide pg. 107.^7^

**TABLE 2:** Scan acquisition parameters for ClearRT determined by selection of mode and FOV

Mode	FOV (mm)	Filtration	Pitch	Couch speed (mm/s)	Nominal IEC Y beam width at isocenter (mm)	Gantry period (s)	Views per rotation
Fine	270	0.5‐mm Cu	0.86	5.24	50	8.22	600
	440	Al bowtie	0.75	4.57			
Normal	440	Al bowtie	0.75	9.11	100	8.28	480
	500	Al bowtie	0.75	9.11			
Coarse	440	Al bowtie	0.75	14.07	140	7.5	360
	500	Al bowtie	0.75	13.41	134		

*Note*: Mode determines the longitudinal beam width, couch speed, and views per rotation, whereas FOV determines filtration.

Abbreviation: FOV, field‐of‐view.

*Source*: Modified from Radixact Physics Essential Guide pg. 108.^7^

In this study, parameter combinations were chosen to assess a range of possible useful clinical protocols at the discretion of the authors as no protocols have yet been established in clinical use. In total, scans were acquired with 10 unique combinations as shown in Table 3 in an effort to characterize the image quality of the system and evaluate how changes to specific parameters impact these metrics. Radiation output as given by reported CTDI_vol_ values for the chosen parameter combinations ranged from 0.5 to 1.4 cGy. No work has yet been performed to evaluate the corresponding patient dose for these images. Scan times for the phantom length of 20 cm were between 25 and 45 s depending on the selection of mode.

**TABLE 3:** ClearRT imaging protocols evaluated in this study

Anatomy	Body size	Mode	mAs	FOV (mm)	Nominal beam width (mm)	Filtration	CTDI_vol_ (cGy)
Head (100 kV)	Medium	Normal	300	270	100	0.5‐mm Cu	0.5
	Medium	Normal	300	440	100	Al bowtie	1.3
Thorax (120 kV)	Small	Normal	192	440	100	Al bowtie	0.6
	Medium	Fine	375	440	50		1.3
	Medium	Normal	300	440	100		0.9
	Medium	Normal	300	500	100		0.8
	Medium	Coarse	225	440	140		0.7
	Large	Normal	384	440	100		1.2
Pelvis (140 kV)	Medium	Normal	300	440	100	Al bowtie	1.3
	Large	Normal	384	500	100		1.4

*Note*: Parameters were chosen to assess a range of possible useful clinical protocols while also evaluating how specific parameters impact image quality.

Abbreviation: FOV, field‐of‐view.

The TomoTherapy MVCT imaging system consists of a conventional linear accelerator mounted opposite to a single‐row xenon ion chamber detector on a continuously rotating ring gantry that acquires axial image slices in a helical pattern as previously described.^8^ The first prototype design for this system featured the idea of a kVCT instead of an MVCT system, but this was dropped for cost considerations and as the work by Fang and Ruchala showed that it was possible to attain MVCT images of higher quality than initially expected.^9^ For imaging, the nominal energy of the incident photon beam is reduced to 3.5 MV to improve image quality and reduce patient dose, whereas the fan beam is collimated to a width of 4 mm with an FOV of approximately 390 mm. The gantry is capable of rotating at up to 6 RPM. The user can select between three scan modes (*Fine*, *Normal*, and *Coarse*) that define the pitch and couch speed. For each mode, two reconstruction intervals are available: *1* and *2 mm* for *Fine, 2* and *4 mm* for *Normal*, and *3* and *6 mm* for *Coarse*. In this study, all possible mode and interval combinations were evaluated. Machine output for these parameters ranged from 0.9 to 2.6 cGy according to CTDI_vol_ values, whereas data from over 20 patient centers showed a typical patient imaging dose of approximately 1–3 cGy for acquisitions with this system.^10^ Note that for the intercomparison of stochastic risk between different imaging scenarios, imaging dose must be converted to effective dose (mSv) as discussed by the American Association of Physicists in Medicine (AAPM) Task Group 75 (TG‐75).^10^

The Radixact MVCT system is nearly identical to its predecessor on TomoTherapy. The main improvement is the inclusion of a new iterative reconstruction (IR) algorithm designed to reduce noise and improve contrast in comparison to the FBP algorithm on TomoTherapy, as demonstrated in a study by Kraus et al.^11^ As previously noted, gantry operation improved to a maximum rotation rate of 10 RPM as well. Available scan parameters are identical to those on the TomoTherapy system, whereas the user is given the ability to select the reconstruction algorithm. Two IR algorithms, *IR General* and *IR Soft Tissue*, as well as the previously used FBP, are available. In this study, all mode and interval combinations were evaluated with an application of the *IR General* reconstruction algorithm. Due to the similarities between the two systems, radiation output and patient imaging dose compare to those described for the TomoTherapy system. Depending on the mode used, scan times for the Radixact and TomoTherapy MVCT systems were 2–5 and 3–6 min, respectively. Importantly, the long scan times (∼5–6 min) for several of these acquisitions are not ideal when attempting to minimize the in‐room time for each patient and maximum patient throughput overall.

The TrueBeam system consists of a kV X‐ray source with an FPD mounted orthogonal to the electronic portal imaging device by means of a robotic arm (Exact), allowing for raw images to be acquired by rotating the gantry over 360° with a maximum gantry rotation rate of 1 RPM.^12^ Two image acquisition modes are available, with *full‐fan* used to image small anatomic sites with an FOV of 240 mm and *half‐fan* used to acquire images over a larger FOV of 450 mm. Full and half bowtie filters were used for both *full* and *half* scans, respectively. For additional specifications of this system, the reader is referred to the TrueBeam specifications manual.^12^ In this study, scans were acquired for four commonly used system presets as shown in Table 4. Machine output for these presets ranged from 0.3 to 1.6 cGy, whereas patient imaging doses in the range of 0.2–4 cGy, depending on acquisition mode and patient size, have been reported in the literature for this imaging system.^13^, ^14^

**TABLE 4:** TrueBeam and Siemens Edge imaging protocols evaluated in this study

System	Acquisition mode	kV	mAs	FOV (mm)	Nominal slice thickness (mm)	Filtration	CTDI_vol_ (cGy)	Scan times
TrueBeam	Head	100	150	240	2.0	Full bowtie	0.3	1–2 min
	Pelvis	125	1050	450		Half bowtie	1.6	
	Thorax	125	260	450		Half bowtie	0.4	
	Spotlight	125	750	240		Full bowtie	1.2	
Siemens Edge	Head	120	350	400	0.6	Standard bowtie	6	<20 s
	Pelvis	120	300	400	3.0		2.0	
	Chest	120	135	500	3.0		0.9	
	Abdomen	120	200	500	3.0		1.3	

*Note*: Protocols were chosen based on clinical relevance. Listed scan times are for the phantom length of 200 mm.

Abbreviation: FOV, field‐of‐view.

The Siemens Edge system is a conventional single‐source multidetector CT system used for CT simulation at this institution. The system is capable of image acquisition at a maximum scan speed of 23 cm/s and a minimum gantry rotation time of 0.28 s. For clinical acquisitions, FOVs of 500 mm for large anatomic sites and 400 mm for head‐and‐neck sites are typically used. All images were acquired with a collimation of 128 mm × 0.6 mm. Images were reconstructed via FBP using an Hr38 kernel with iMAR for head acquisitions and a Br38 kernel without iMAR for all other body sites. For further information on system specifications, the reader is referred to the system brochure.^15^ In this study, scans were acquired for clinically relevant protocols at the discretion of the authors as shown in Table 4. Although no imaging doses were found reported in the literature, machine output in this study ranged from 0.9 to 6.1 cGy depending on acquisition mode based on reported CTDI_vol_ values.

### 2.2 Image quality phantom

**FIGURE 1:**
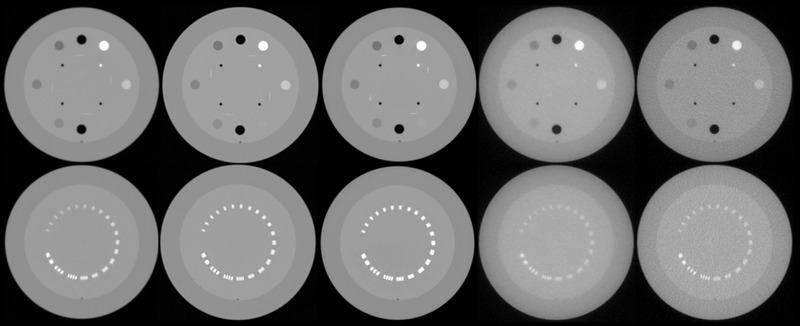
Axial image slices of phantom modules CTP404 (top row) and CTP528 (bottom row) for CT scans acquired in this study for (L–R) ClearRT with *Thorax* anatomy, *Medium* body size, *Fine* mode, 440‐mm FOV; Siemens Edge with *Pelvis* anatomy, 400‐mm FOV; TrueBeam OBI kV CBCT with *Pelvis* anatomy, 450‐mm FOV; Radixact MVCT with *Fine* mode, *1‐mm* reconstruction interval, ∼390‐mm FOV; TomoTherapy MVCT with *Fine* mode, *1‐mm* reconstruction interval, ∼390‐mm FOV. CBCT, cone‐beam CT; CT, computed tomography; FOV, field‐of‐view

The CatPhan 504 (The Phantom Laboratory, Inc., Greenwich, NY) phantom was used to evaluate image quality. Though originally developed for use with kVCT systems, it was utilized across all systems to maintain consistency throughout the study. The phantom has a diameter of 20 cm as recommended by the report of the AAPM TG‐12 and is composed of several modules for a complete characterization of imaging performance for axial and spiral CT scanners.^16^ Module CTP486, used for noise and uniformity evaluations, is cast from uniform materials designed to be within 2% of water's density for standard scanning protocols. CTP404, used for CT linearity evaluation, consists of cylindrical inserts of seven materials with known densities: air, polymethyl pentene, low‐density polyethylene (LDPE), polystyrene, acrylic, Delrin, and Teflon. Several inserts of this module were also used for the measurement of contrast metrics. Lastly, CTP528 was used for spatial resolution analysis and consists of a 1–21 line pairs per centimeter (lp/cm) test gauge for high‐resolution evaluation. For each acquisition, the phantom was positioned according to vendor specifications.^17^ Figure 1 shows axial image slices of phantom modules CTP404 (top row) and CTP528 (bottom row) for each of the imaging systems evaluated.

### 2.3 Implementation

Data analysis was performed using ImageJ software (National Institutes of Health, USA) with the exception of the spatial resolution evaluation. For each acquisition, the appropriate number of slices (based on slice thickness) was evaluated to ensure that a similar scan extension of approximately 15–18 mm in each module was analyzed for all images. Each individual slice was evaluated for artifacts that may have affected image quality measurements before analysis was performed.

### 2.4 Image quality metrics

#### 2.4.1 Noise

Image noise, defined as the standard deviation of pixel values within some homogenous region of interest (ROI), was assessed by placing five ROIs, approximately 1% of the phantom area in size, at the center of the image slices and four periphery locations roughly one ROI diameter from the edge of the measurement area in CTP486, as detailed by AAPM TG‐233.^18^ As slight “cupping” or “capping” artifact was present in all images, it was determined that use of one large ROI as recommended by AAPM TG‐12 would have posed the complication of decoupling noise and image nonuniformity. The mean pixel values and the standard deviation in these values were measured for each ROI to calculate image noise, which was averaged across all five ROIs. Noise was reported as the standard deviation of pixel values and as the corresponding percentage of the mean pixel value (i.e., as the uncertainty in the mean pixel value) and averaged over all image slices for each acquisition.

#### 2.4.2 Uniformity

Image uniformity was assessed utilizing the same ROIs described earlier. For each image slice, the uniformity index (UI) describing the amount of “cupping” or “capping” artifact was calculated as defined in the following equation:

(1) $$UI\;=\;\mu_{CT,\;P}-\;\mu_{CT,\;C}$$

where $\mu_{CT,\;P}$ and $\mu_{CT,C}$ are the mean CT numbers of the periphery and center ROIs. Note that for Equation (1), a positive value indicates a “cupping” artifact (periphery ROIs appear “brighter” than the center ROI), whereas a negative value indicates a “capping” artifact (center ROI appears brighter than the periphery ROIs). A “cupping” artifact is a result of beam hardening, as the exit beam energy is higher for rays going through the center of the phantom compared to those going through the edge due to the preferential attenuation of low‐energy X‐rays. Therefore, the transmission through the center is “higher” than expected and the linear attenuation coefficient, the basis for CT number measurement, appears lower. The “capping” artifact is often a result of software overcorrection to account for “cupping.”

Integral nonuniformity (INU), which describes the maximum deviation from the mean pixel value, was also calculated for each slice according to the phantom vendor as seen in the following equation:

(2) $$INU\;=\;\frac{\mu_{PV,Max}-\;\mu_{PV,\;Min}}{\mu_{PV,Max}+\;\mu_{PV,Min}}$$

where $\mu_{PV,Max}$ and $\mu_{PV,Min}$ are the maximum and minimum mean pixel values across all ROIs. Both UI and INU values were averaged over all slices for each acquisition. For each metric, a value closer to zero indicates a more uniform image.

#### 2.4.3 Contrast

Two metrics were used to assess contrast performance with CTP404. Low‐contrast visibility (LCV) refers to the ability to distinguish between materials with similar attenuation properties. Due to their similar mass densities, this metric was calculated using the polystyrene and LDPE inserts as defined in the following equation from Lehmann et al.^19^:

(3) $$LCV\;=\;2.75\times \frac{\sigma_{PV,\;Poly}+\;\sigma_{PV,LDPE}}{\mu_{PV,Poly}-\;\mu_{PV,LDPE}}$$

where $\sigma_{PV,Poly}$ and $\sigma_{PV,LDPE}$ are the standard deviations in the mean pixel values within the inserts, and $\mu_{PV,Poly}$ and $\mu_{PV,LDPE}$ are the mean pixel values within the inserts.

The contrast‐to‐noise ratio (CNR), a description of the signal intensity difference of two regions scaled to the noise, was evaluated for the polystyrene and Delrin inserts according to the following equation from Stützel et al.^20^:

(4) $$CNR\;=\;\frac{\left|\mu_{CT,Insert}-\;\mu_{CT,BG}\right|}{\sqrt{\sigma_{Insert}^{2}+\;\sigma_{BG}^{2}}}$$

where $\mu_{CT,Insert}$ and $\mu_{CT,BG}$ are the mean CT numbers for the ROI in the insert and an ROI in the background region near the insert, and $\sigma_{Insert}$ and $\sigma_{BG}$ are the standard deviations in these values. As before, contrast metrics were averaged over all slices for each acquisition. Lower LCV values indicate an increased ability to distinguish between materials with similar electron densities, whereas higher CNR values indicate an improved signal intensity difference between two different materials.

#### 2.4.4 CT number linearity

The accuracy of CT numbers when compared to nominal values becomes important when scans are used for dose calculations.^1^ Additionally, CT images are a reconstruction of linear attenuation coefficients in each voxel. “Linearity” is a property of a system characterized by an output that is directly proportional to the input. In CT, this property describes the amount to which the CT number of some material is exactly proportional to the linear attenuation coefficient and can be utilized to describe the performance of the system. As a result, the mean CT numbers for each insert in CTP404 were plotted against the linear attenuation coefficient of each insert provided by the vendor to observe and analyze the linear fit of the data. As linear attenuation coefficients vary with energy, the value at the estimated effective energy of each beam was utilized for these plots with the assumption that the effective energy is roughly one‐half the nominal energy. In addition, the variations of mean CT number values among various ClearRT acquisitions were analyzed to provide an indication of how protocol‐specific the calibration curves (CT number vs. relative electron density) may need to be to accurately describe the density of a given scanned material.

#### 2.4.5 Spatial resolution

The spatial resolution of each system was evaluated by the system modulation transfer function (MTF), which is the Fourier transform of the line spread function for a linear, shift‐invariant system and describes contrast recovery as a function of the spatial frequency. The MTF was measured with Python code utilizing the *Pylinac* library designed specifically for image quality assurance tests outlined by AAPM TG‐142.^21^ A collapsed circle profile was extracted about the line pairs in CTP528 to analyze peaks and valleys of each profile to derive the MTF, which was normalized to the first line pair. Only slices with the most easily visible line pairs were evaluated. The MTF values at both 50% (MTF_50%_) and 10% (MTF_10%_) of the original contrast value were reported with units of cycles per millimeter (cycles/mm).

## 3 RESULTS

For the kVCT systems, there was a discernable difference in several image quality metrics between certain small (head and neck) and large anatomy (pelvis and thorax/chest) protocols. As such, results are reported for small and large anatomy protocols for the kVCT systems for a more objective comparison, whereas averages across all acquisition modes for the MVCT systems are reported for all metrics except spatial resolution (as only *Normal* mode was used for this evaluation). However, two of the corresponding figures do not discriminate between anatomy types due to a limited number of acquisitions for head protocols for each kVCT system (Figures 3 and 4). Note that “small” anatomy refers only to head and neck in this study. As the head is a unique site to image due to soft tissue encased in the skull, these protocols may vary significantly from other “small” anatomy protocols (e.g., the knee).

### 3.1 Noise

Image noise for all systems is shown in Table 5. For each kVCT system, noise was higher on average for small anatomy protocols than large anatomy protocols. Overall, noise was the lowest for Siemens Edge though values for large anatomy protocols were similar for ClearRT. For the MVCT systems, the application of the IR algorithm on the Radixact system decreased noise by over a factor of 2.5 when compared to the FBP algorithm on the TomoTherapy system despite other components of the imaging systems remaining nearly identical. However, noise for the Radixact system was still higher by roughly a factor of 2 when compared to large anatomy protocols for ClearRT and Siemens Edge.

**TABLE 5:** Image noise for each system

System	Image noise ($\sigma_{PV})$	Image noise (%)
ClearRT kVCT	8.6 ± 2.1^a^	0.83 ± 0.13^a^
	6.0 ± 1.3^b^	0.56 ± 0.11^b^
TrueBeam kV CBCT	27.7 ± 0.1^a^	2.68 ± 0.05^a^
	8.1 ± 2.5^b^	0.79 ± 0.18^b^
Siemens Edge kVCT	6.5 ± 0.1^a^	0.62 ± 0.01^a^
	5.5 ± 1.1^b^	0.53 ± 0.11^b^
Radixact MVCT	12.9 ± 0.1	1.18 ± 0.01
TomoTherapy MVCT	32.5 ± 0.2	2.99 ± 0.01

*Note*: Noise is reported as the standard deviation in the mean pixel value and as a percentage of the mean pixel value, averaged across all acquisitions. Values are reported to one standard deviation across all measurements of the same protocol type. Note that in this study, “small” anatomy refers only to head and neck.

Abbreviation: CBCT, cone‐beam computed tomography.

^a^ Small anatomy protocols.

^b^ Large anatomy protocols.

**FIGURE 2:**
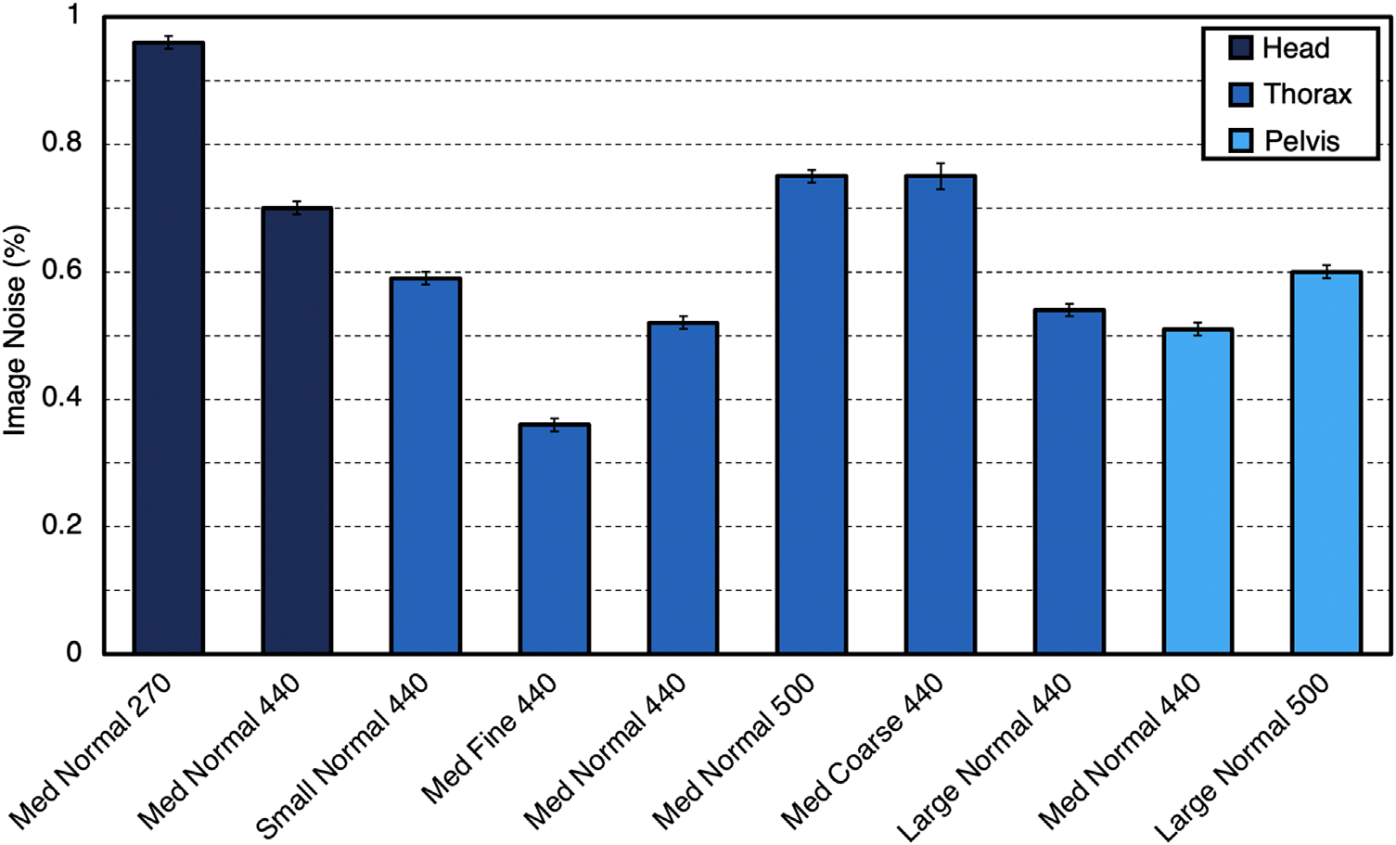
Noise as a percentage of the mean pixel value for each ClearRT protocol in this study. Error bars represent one standard deviation. Protocol labels are *body size‐mode‐FOV*. FOV, field‐of‐view

Noise for individual ClearRT protocols is shown in Figure 2. For ClearRT acquisitions, noise was reduced when changing from *Coarse* to *Fine* mode (assuming that all other parameters remained identical) as mAs was subsequently increased (due to an increase in views per rotation) and the nominal longitudinal beam width decreased leading to a reduction in scatter. A slight reduction in noise was also seen when changing from *Head* to *Pelvis* anatomy for identical parameters.

### 3.2 Uniformity

**FIGURE 3:**
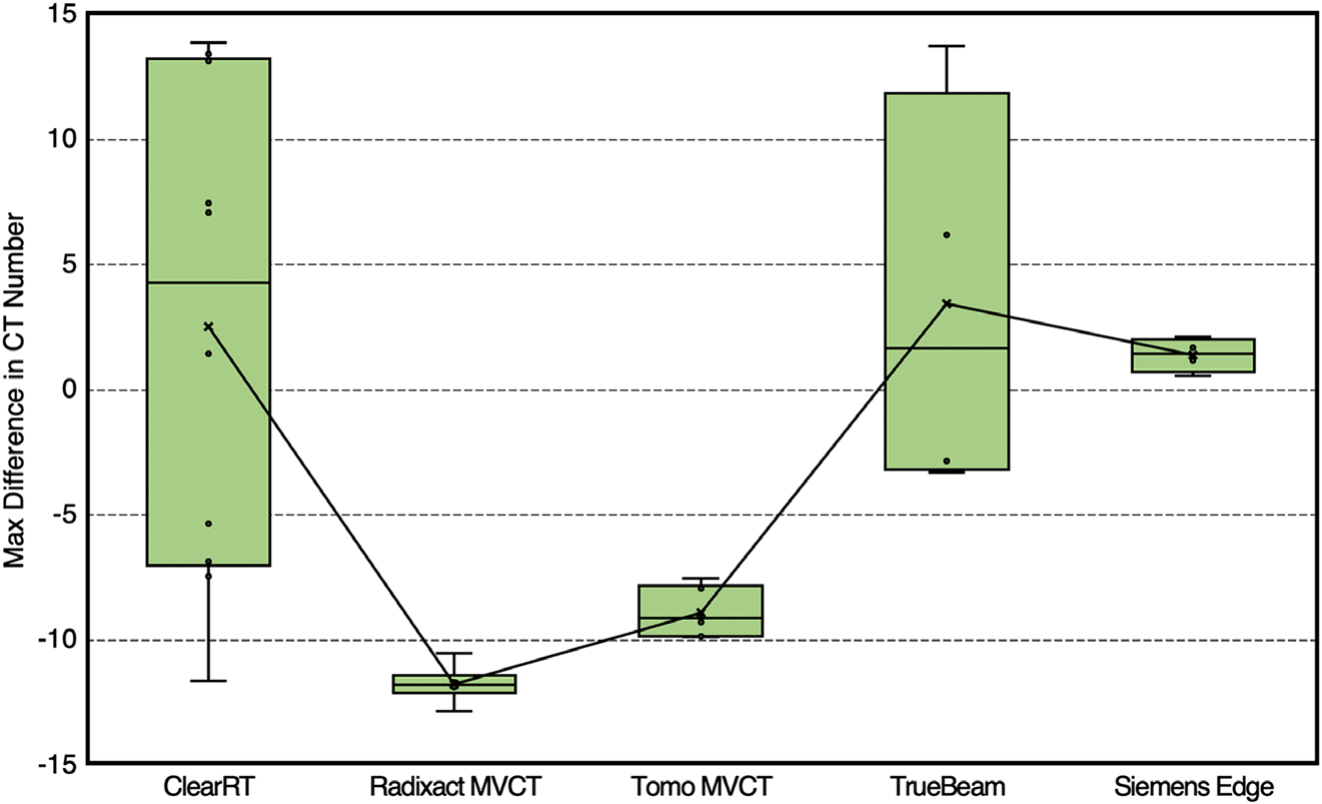
UI values across all acquisition protocols for each system. Values close to zero indicate high image uniformity, whereas positive or negative values indicate the presence of “cupping” or “capping” artifact, respectively. The bars represent the maximum and minimum values, whereas the upper and lower values of the box plot represent quartile 3 (75th percentile) and quartile 1 (25th percentile), respectively. The median value is given by the line within the box. Additionally, mean values are shown with an “x” and lines connecting the mean values are plotted. UI, uniformity index

**FIGURE 4:**
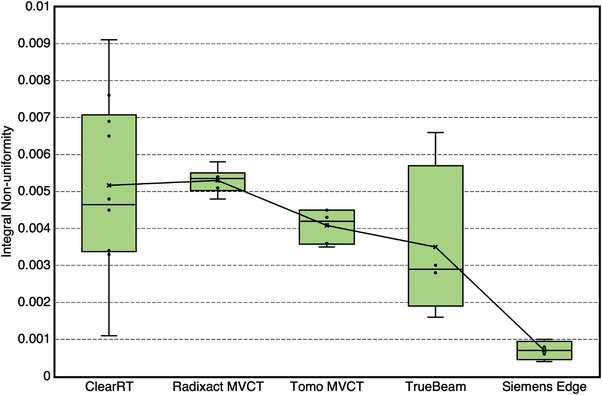
INU values across all acquisition protocols for each system. Values close to zero indicate little variation in the mean pixel values between regions of interest in a homogenous phantom module. The bars represent the maximum and minimum values, whereas the upper and lower values of the box plot represent quartile 3 (75th percentile) and quartile 1 (25th percentile), respectively. The median value is given by the line within the box. Additionally, mean values are shown with an “x” and lines connecting the mean values are plotted. INU, integral nonuniformity

The variability of UI and INU values across all acquisitions for each system is seen in Figures 3 and 4. Individual UI values for ClearRT and TrueBeam indicate roughly half the scans exhibited a “cupping” artifact (positive value), whereas the others exhibited a “capping” artifact (negative value). All Siemens Edge scans exhibited a slight “cupping” artifact, whereas all MVCT scans demonstrated a relatively significant “capping” artifact in comparison to the kVCT systems. However, though the magnitudes of UI values were largest for the MVCT systems, their INU values generally indicated less variability in uniformity across the entire image than ClearRT. Overall, the most uniform images were produced by the Siemens Edge system. For the kVCT systems, large anatomy scan protocols showed better overall uniformity than small anatomy scan protocols when considering both metrics.

**FIGURE 5:**
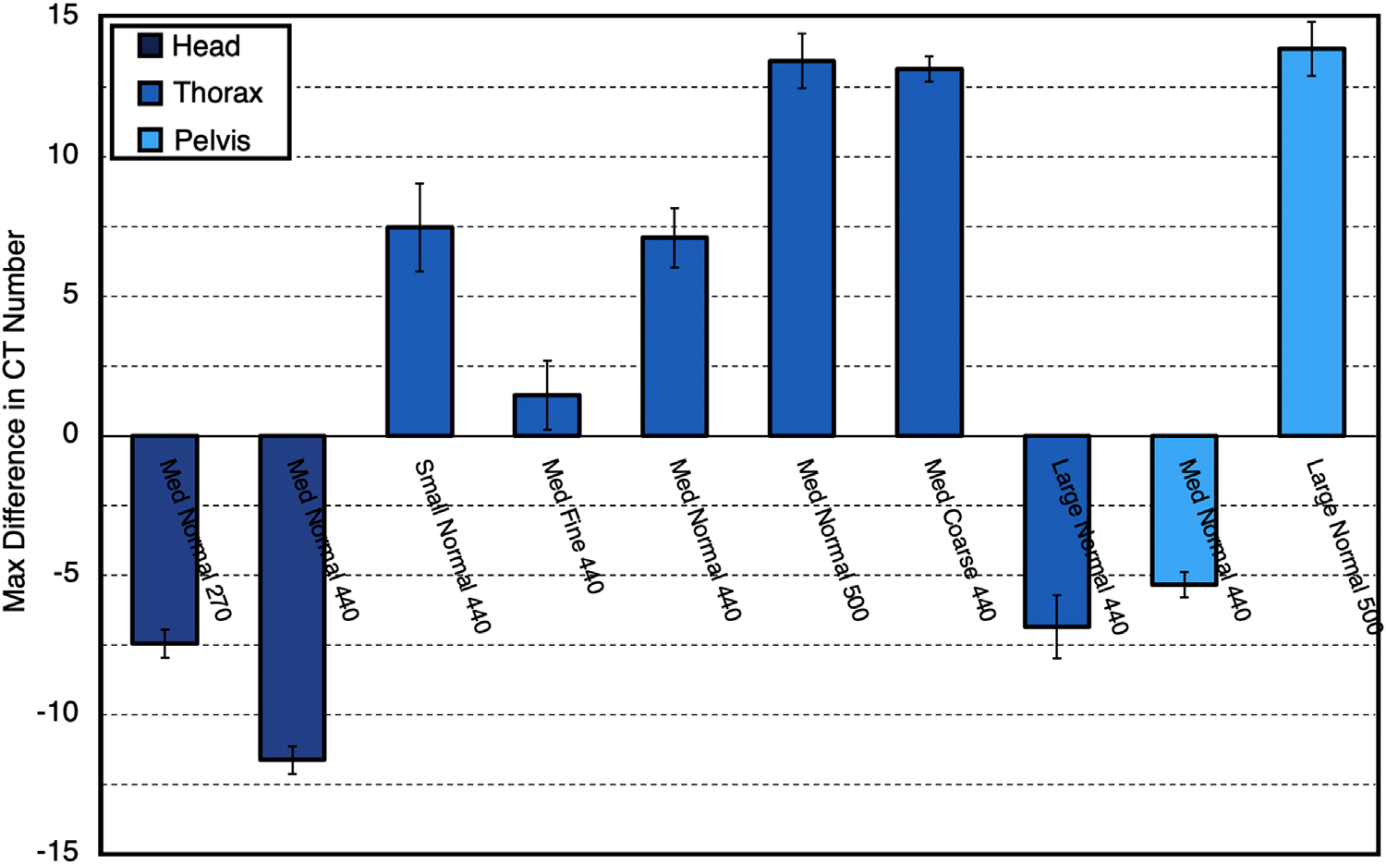
UI values for each ClearRT protocol in this study. Values close to zero indicate high image uniformity, whereas positive or negative values indicate the presence of “cupping” or “capping” artifact, respectively. Error bars represent one standard deviation. Protocol labels are *body size‐mode‐FOV*. FOV, field‐of‐view; UI, uniformity index

**FIGURE 6:**
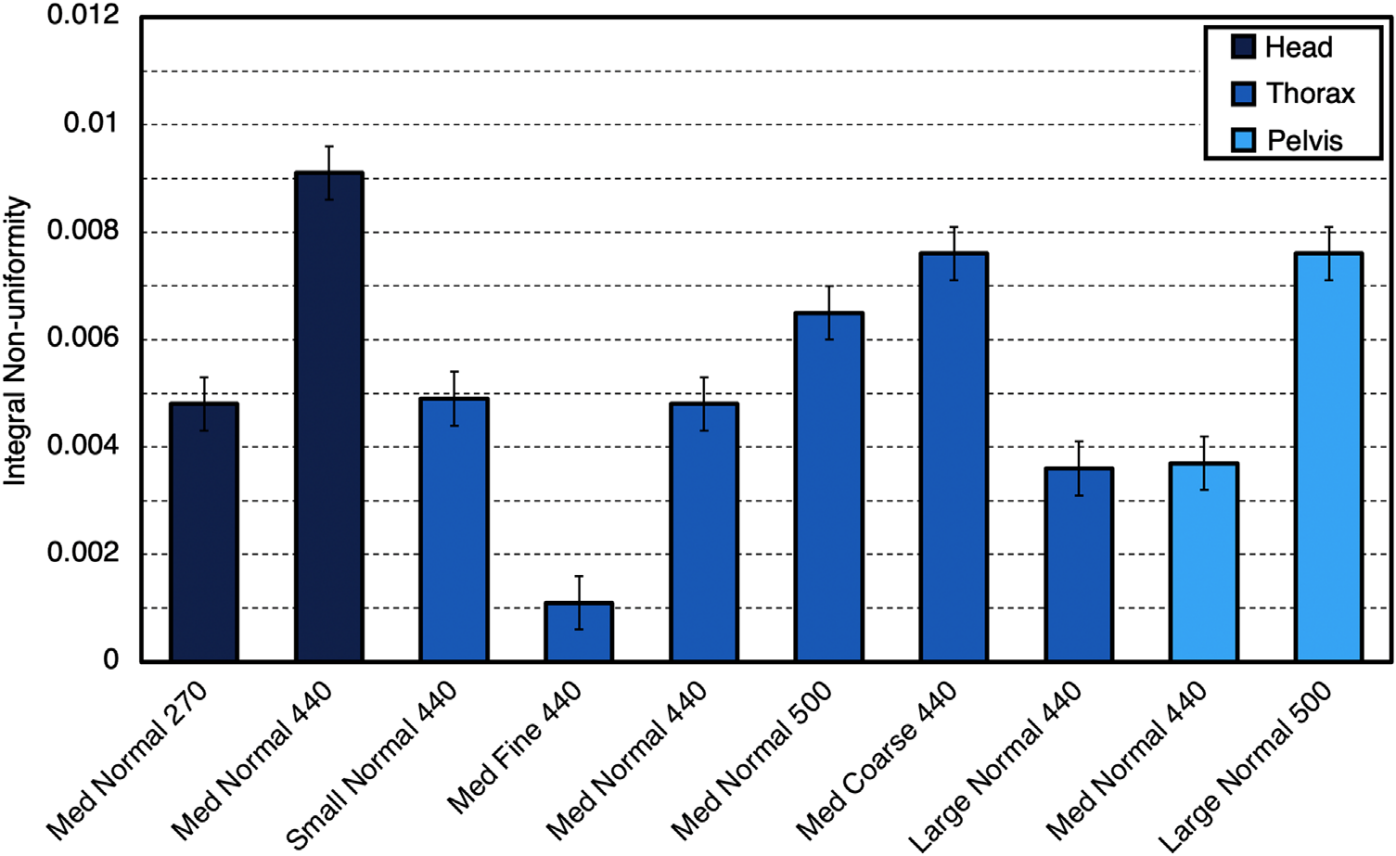
INU values for each ClearRT protocol in this study. Values close to zero indicate little variation in the mean pixel values between regions of interest in a homogenous phantom module. Error bars represent one standard deviation. Protocol labels are *body size‐mode‐FOV*. FOV, field‐of‐view; INU, integral nonuniformity

UI and INU values for each individual ClearRT protocol are shown in Figures 5 and 6. Uniformity was improved when varying the mode from *Coarse* to *Fine* and as the nominal beam energy was increased. An increase in FOV for ClearRT reduced image uniformity for each anatomy assuming all other parameters remained identical.

### 3.3 Contrast

**FIGURE 7:**
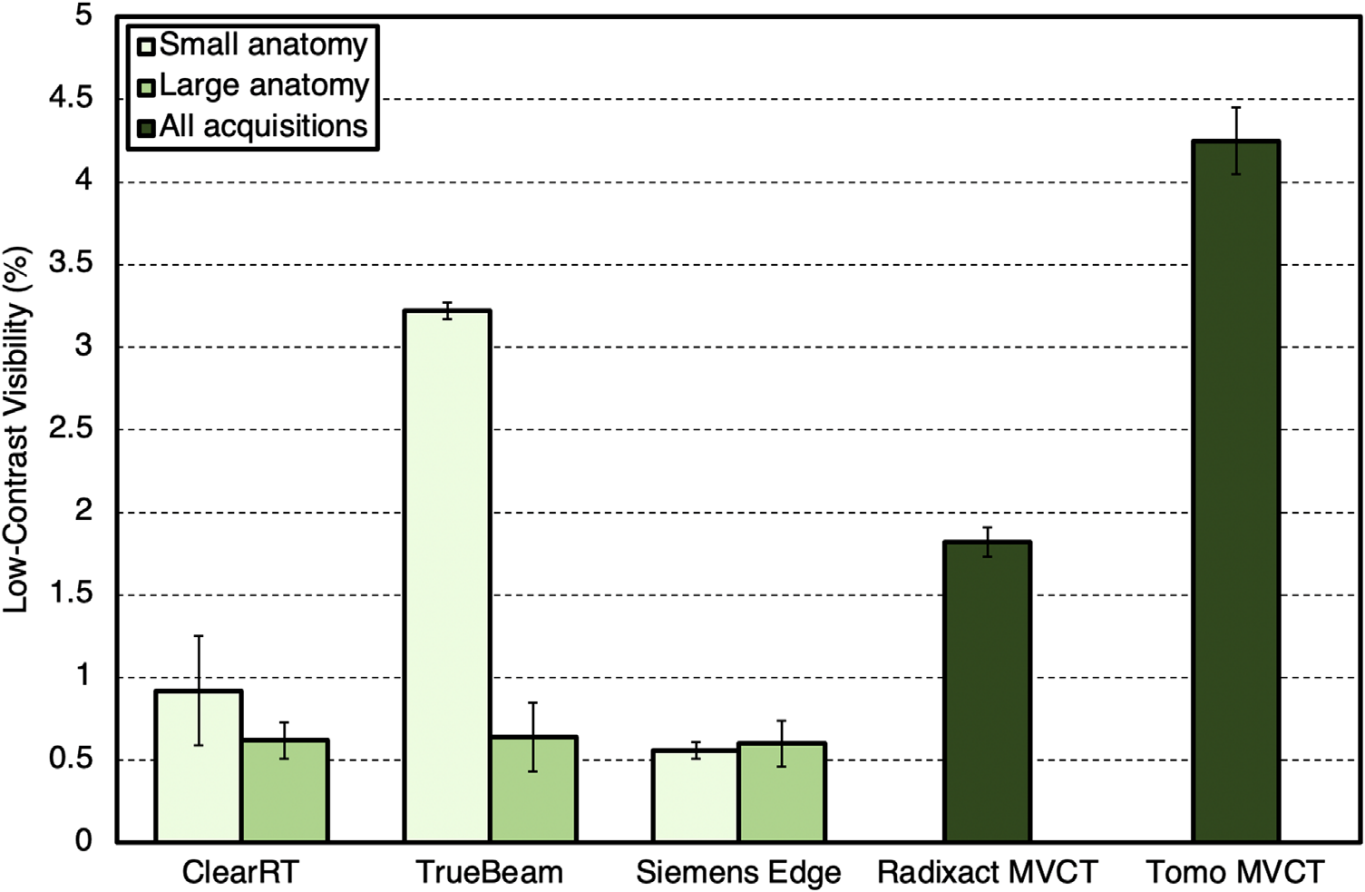
LCV values for each system. Values are reported as an average across all acquisitions for the MVCT systems and separated into small and large anatomy protocols for the kVCT systems. A lower value indicates an increased ability to distinguish between materials with similar attenuation properties. Error bars represent one standard deviation. Note that in this study, “small” anatomy refers only to head and neck. LCV, low‐contrast visibility

**FIGURE 8:**
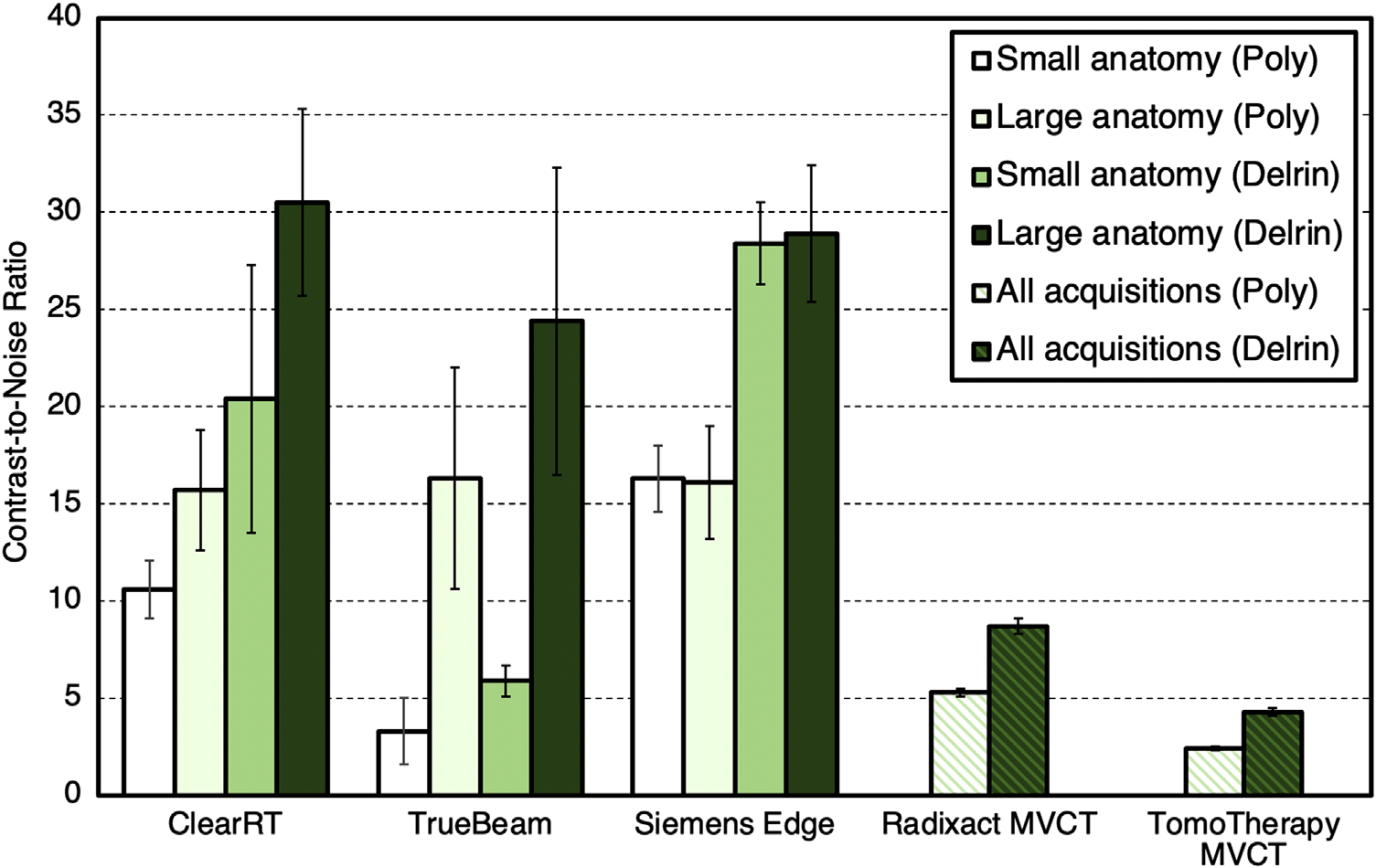
CNR values for each system and each inset used in the analysis. Values are reported as an average across all acquisitions for the MVCT systems and separated into small and large anatomy protocols for the kVCT systems. Error bars represent one standard deviation. Note that in this study, “small” anatomy refers only to head and neck. CNR, contrast‐to‐noise ratio

Values for LCV are shown in Figure 7. For ClearRT and TrueBeam, values for LCV were higher for small anatomy protocols compared to large anatomy protocols on average. For the Siemens Edge system, there was no discernable difference between anatomy types. For large anatomy protocols, the kVCT systems produced similar values for LCV across all acquisitions. For CNR evaluation of the polystyrene insert, values for small anatomy protocols were reduced when compared to large anatomy protocols for ClearRT and TrueBeam, whereas for Siemens Edge, no discernable difference was observed between anatomy types once again. For the Delrin insert, CNR values were also increased for large anatomy protocols for ClearRT and TrueBeam, whereas these values were similar for Siemens Edge as seen in Figure 8. For the MVCT systems, Radixact once again showed improvement over TomoTherapy as LCV and the CNR values for each insert evaluated were improved by at least a factor of 2.

**FIGURE 9:**
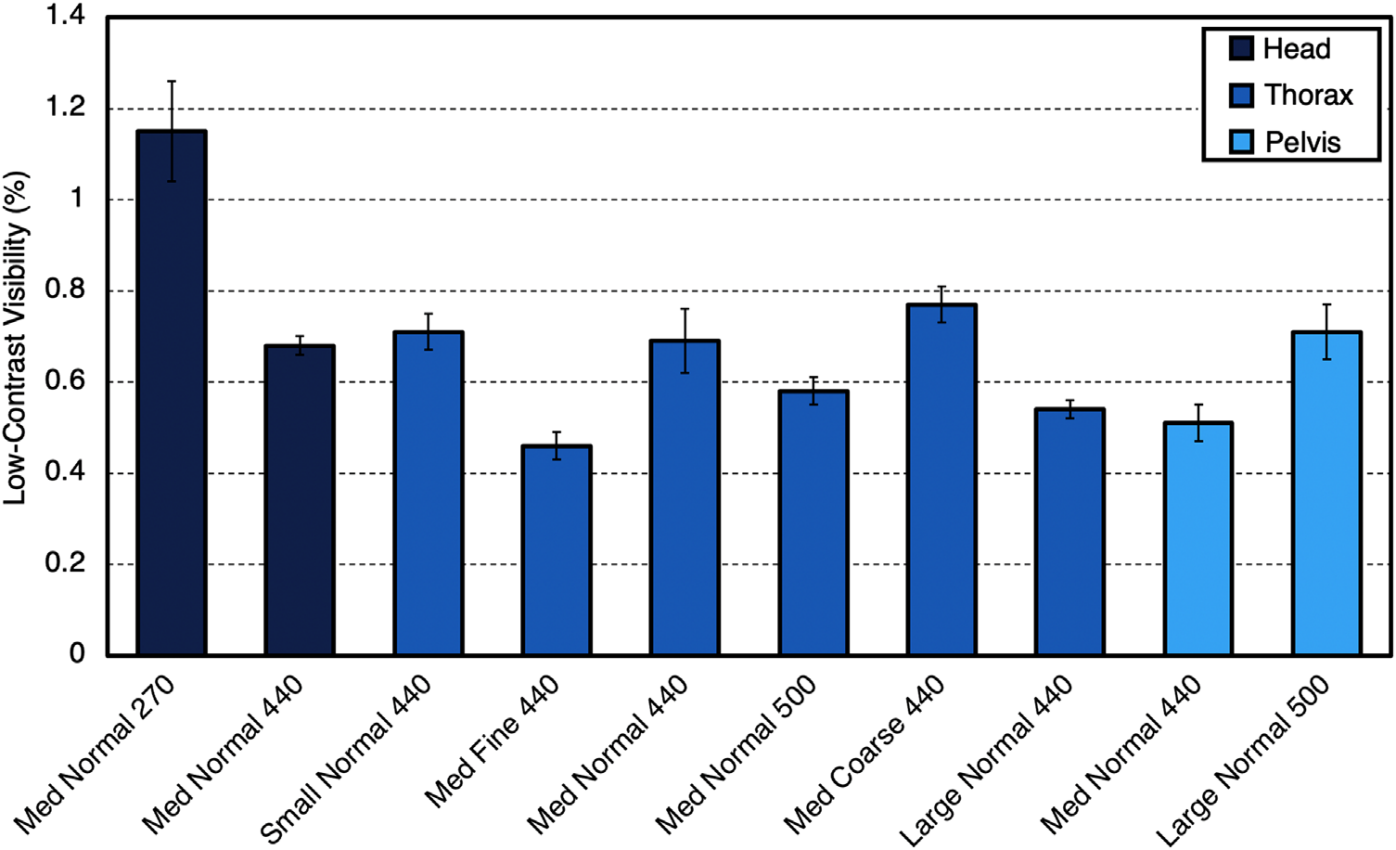
LCV values for each ClearRT protocol in this study. A lower value indicates an increased ability to distinguish between materials with similar attenuation properties. Error bars represent one standard deviation. Protocol labels are *body size‐mode‐FOV*. FOV, field‐of‐view; LCV, low‐contrast visibility

**FIGURE 10:**
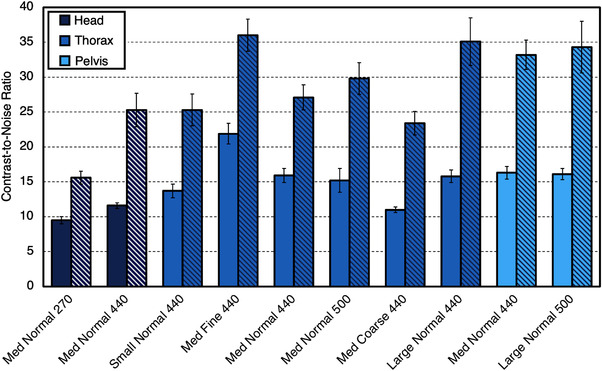
CNR values for each ClearRT protocol in this study and each inset used in the analysis. Values for the polystyrene insert are shown with solid colors, whereas values for the Delrin insert are shown with striated colors. Error bars represent one standard deviation. Protocol labels are *body size‐mode‐FOV*. CNR, contrast‐to‐noise ratio; FOV, field‐of‐view

LCV and CNR values for each individual ClearRT protocol can be seen in Figures 9 and 10. The selection of the *Fine* protocol on ClearRT improved the values of these contrast metrics as before due largely to a decrease in image noise as previously described.

### 3.4 CT number linearity

**FIGURE 11:**
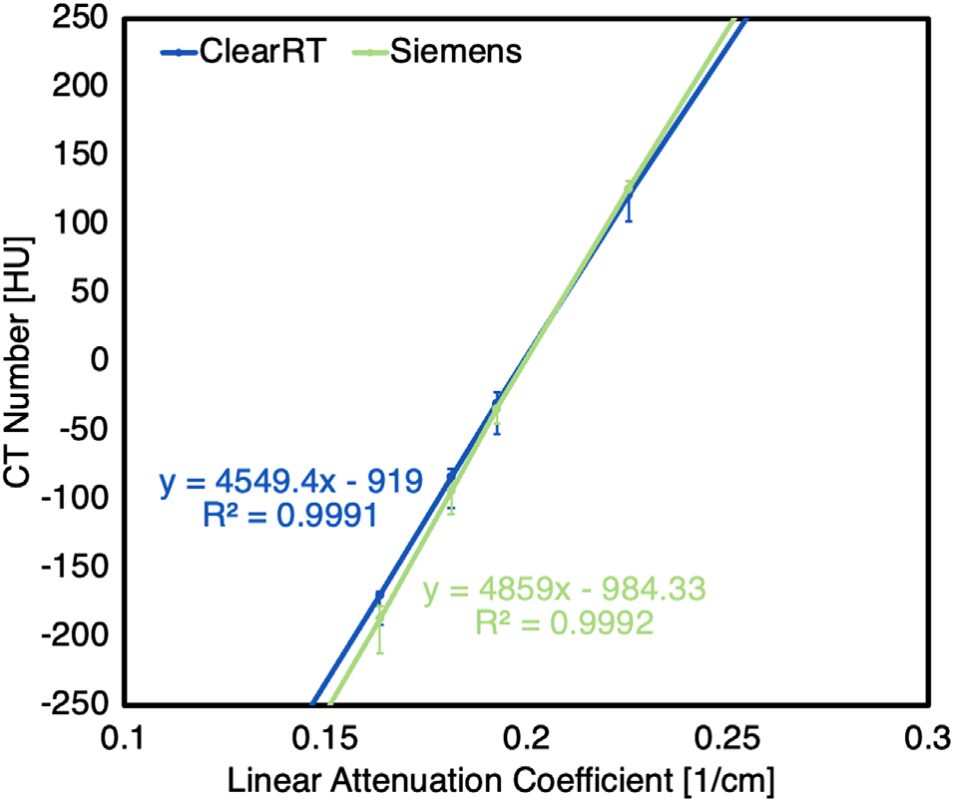
Linear attenuation coefficient plotted against mean CT numbers at 120 kV for the ClearRT and Siemens Edge. These curves were constructed using mean CT numbers across all acquisitions at this nominal energy. Error bars represent maximum and minimum CT numbers across these acquisitions. *R* ^2^ values indicate the linear fit of the data with the equation for the linear fit also provided. CT, computed tomography

The MVCT systems showed good linearity when CT numbers were plotted against linear attenuation coefficients for each insert in CTP404. A similar level of linearity was achieved between each kVCT system, though this linearity was slightly reduced when compared to the MVCT systems. For ClearRT, *R* ^2^ values for the linear fit for small and large anatomy protocols were 0.9986 ± 0.0020 and 0.9993 ± 0.0011, respectively, whereas values for small and large anatomy protocols for TrueBeam and Siemens Edge were 0.9973 ± 0.0006 and 0.9988 ± 0.0005 and 0.9956 ± 0.0005 and 0.9985 ± 0.0005, respectively. *R* ^2^ values of 1.0000 were seen for all MVCT acquisitions. Mean CT numbers of each insert were also more consistent across acquisitions (for the same nominal beam energy) for the MVCT systems. Figure 11 shows the curve of linear attenuation coefficients plotted against average CT numbers for the inserts. For five of the seven inserts (at this energy), the maximum difference in mean CT numbers between protocols was lower for ClearRT than Siemens Edge.

### 3.5 Spatial resolution

Though a slight difference was observed between small and large anatomy protocols for ClearRT and TrueBeam, spatial resolution varied little across all acquisition modes for each system regardless of scan parameters. Resolution was best for TrueBeam and Siemens Edge, based on MTF_50%_ and MTF_10%_ values. ClearRT and TomoTherapy showed similar spatial resolution, with MTF_50%_ and MTF_10%_ values reduced from TrueBeam and Siemens Edge by approximately 15% and 10%, respectively, as shown in Table 6. Resolution was the lowest for Radixact MVCT.

**TABLE 6:** Relative MTF values for each system

System	MTF 50% (mm^−1^)	MTF 10% (mm^−1^)
ClearRT kVCT	0.28 ± 0.02^a^	0.44 ± 0.06^a^
	0.25 ± 0.01^b^	0.40 ± 0.01^b^
TrueBeam kV CBCT	0.33 ± 0.01^a^	0.49 ± 0.01^a^
	0.30 ± 0.01^b^	0.45 ± 0.01^b^
Siemens Edge kVCT	0.29 ± 0.01^a^	0.48 ± 0.01^a^
	0.29 ± 0.03^b^	0.46 ± 0.03^b^
Radixact MVCT	0.22 ± 0.02	0.38 ± 0.03
TomoTherapy MVCT	0.26 ± 0.03	0.44 ± 0.01

*Note*: Values are normalized to the first line pair in phantom module CTP528 and reported as an average across all Normal mode acquisitions for the MVCT systems and separated into small and large anatomy protocols for the kVCT systems. Note that in this study, “small” anatomy refers only to head and neck.

Abbreviations: CBCT, cone‐beam computed tomography; MTF, modulation transfer function.

^a^ Small anatomy protocols.

^b^ Large anatomy protocols.

## 4 DISCUSSION

The purpose of this work was to compare the image quality of a novel helical kVCT system to that of CT systems established clinically for use in IGRT and/or ART (dose calculation). Although the quality of the individual components of each system and variation in imaging parameters such as FOV and slice thickness impact overall image quality, the objective of this study was to evaluate image quality under simulated clinical settings for each system as a whole with a focus on relative comparison across systems and within the ClearRT system itself. As such, although resampling the data to match imaging parameters such as FOV and slice thickness would have provided equivalent resolution for the metric calculations within the image sets, this would not represent a common modification in clinical practice. Additionally, as ClearRT was only recently approved for clinical use, scan protocols were chosen in an attempt to evaluate this system for a wide range of possible clinical settings to assess how the selection of certain parameters impacts image quality. It is possible that several of the chosen protocols will not prove useful in the clinic. As a result, more variability was generally seen across measurements for this system, resulting in the possibility of poorer values on average for several metrics than if different protocols had been used. The authors also acknowledge that phantom size may have played a role in the difference in image quality between small and large anatomy protocols for certain metrics for the kVCT systems, as systems generally adjust the fluence of the beam (and energy) as appropriate based on the anatomy to be imaged. As the CatPhan 504 is more representative of a head phantom, the use of a larger phantom for large anatomy protocols would likely have degraded image quality to some extent compared to what was reported. As such, the quantitative values provided in this study are representative of the phantom utilized and are not absolute. However, the use of a single phantom allowed for an objective comparison across imaging systems and between individual ClearRT protocols. The application of different filters for different field sizes within the *Head* protocol may also have played a role in these differences for ClearRT. The authors also emphasize that these results are representative of systems available for use at this institution and note that image quality may vary between each individual system of the same make and model. Finally, acquisitions for the ClearRT system were taken with the latest system and software versions available as of the spring of 2021, whereas system and software versions for the other systems were up‐to‐date as of winter/spring 2020–2021. As such, any future system and/or software updates may possibly yield altered values for several of these metrics.

Prior to discussion of the results, it should first be stated that measured values for these metrics for all systems were within tolerances specified by the manufacturers when applicable. Overall, Siemens Edge outperformed the other systems when considering all image quality metrics; however, this system was designed to produce diagnostic‐quality images for use in CT simulation, and thus this result is not unexpected. ClearRT performed well in comparison for metrics most important to IGRT, mainly noise and LCV. Although the TrueBeam and Siemens Edge showed improved spatial resolution when compared to ClearRT, the slight reduction in values for ClearRT is not expected to impact its fidelity for IGRT and/or ART implementation in any appreciable manner.

When considering image quality, it is also important to consider the cost of added dose from these acquisitions. Daily imaging doses are small when compared to therapeutic doses; however, these doses are spread over the entire volume being imaged, a majority of which is often healthy tissue, as opposed to concentration at a target volume. It is not uncommon for additional accumulated doses of 3–370 cGy to occur over the entire course of a treatment, which exceeds the threshold doses reported in the literature for secondary malignancy to occur as discussed by AAPM TG‐179.^1^, ^22^, ^23^ Although no work has yet been performed to analyze patient dose for the ClearRT system, the reported CTDI_vol_ values provide a metric by which to directly compare the output of these systems, which roughly mimic patient dose. Overall, output for ClearRT was comparable to Siemens Edge for large anatomy protocols and significantly reduced by a factor of 5–10 for the small anatomy protocols. It is important to emphasize once more that “small” anatomy refers only to head and neck in this study, as other “small” anatomy extremity protocols for Siemens Edge would have a reduction in mAs. Output for ClearRT was also generally lower compared to the MVCT systems except when *Coarse* mode was applied for the MV systems (note that for the MV systems mode did not seem to have a significant bearing on image quality for a stationary phantom). Each facility should discuss the cost of extra imaging dose versus treatment benefits (including improved image quality and patient setup) offered by these images with the medical physicists and radiation oncologists to reach a compromise that best suits the goals of the treatment to optimize patient benefit.^10^

The impact of parameter selection on image quality for ClearRT is summarized in Table 7. The selection of mode most impacts image quality assuming all other parameters remain identical. Images acquired using *Fine* mode were of the highest quality as noise was reduced, and contrast and uniformity were improved compared to the other modes. This is due to the large reduction in nominal beam width (50 mm) for *Fine* mode and the subsequent increase in views per rotation (and thus mAs). Using *Normal* (100 mm) or *Coarse* (∼140 mm) mode introduces more of a cone‐beam‐type geometry, and CBCT has been seen to be more susceptible to scattering, beam hardening, and artifacts that degrade image quality.^24^ However, the selection of *Fine* mode comes at the cost of additional patient dose as radiation output is increased by a factor of approximately 2 when compared to identical acquisitions under *Coarse* mode. Nonetheless, this output may still be similar or reduced relative to the other systems depending on the additional parameters chosen. Also, the selection of *Fine* mode increases the imaging time by nearly a factor of two when compared to *Coarse* mode. However, in this study, scan times were still on the order of seconds regardless of the mode used. For large anatomy protocols on ClearRT, an increase in FOV was observed to increase noise and decrease uniformity overall while decreasing machine output. For the *Head* protocol, increasing the FOV from 270 to 440 mm greatly improved image quality while increasing machine output by a factor of 2–3 depending on the mode. This is likely (at least partly) due to the fact that changing from a 270‐ to 440‐mm FOV for *Head* anatomy involves changing from a 0.5‐mm Cu filter to an aluminum bowtie filter. Analysis of half‐value layers (HVLs) measured by the authors for this system showed that beams for *Head* protocols have a much lower HVL for the bowtie filter (∼6.5‐mm Al) when compared to the 0.5‐mm Cu filter (∼9.0‐mm Al), meaning a softer beam consisting of lower energy X‐rays (and a subsequent increase in superficial patient dose). Thus, one drawback of using the larger FOV in the clinical setting would be a higher patient dose based on reported CTDI_vol_ values. Additionally, though this softer beam improved image quality for the phantom used, in a clinical head case, it may prove beneficial to use the harder beam due to beam interaction within the relatively dense skull. The selection of larger body sizes slightly improves image quality due to an increase in mA per view (mAs). It is expected that the impact on image quality for the increase (or decrease) of mAs due to the selection of body size will be more evident in a larger phantom. Finally, slight improvement in image quality was also observed as the nominal beam energy increased (due to the selection of anatomy). Once again, it is expected that observed differences in image quality due to differences in the nominal beam energy will be more evident as phantom (or patient) size increases.

**TABLE 7:** Impact of parameter selection on image quality and scan time for ClearRT

Parameter	Modification	Impact on image quality	Impact on machine output	Impact on acquisition time
Anatomy	Head → Pelvis	Noise, uniformity, and contrast slightly improved	Limited (energy is increased)	None
Body size	Small → Large	Noise, uniformity, and contrast slightly improved	Increased by up to a factor of 2	None
Field‐of‐view	Increase	Improved image quality for *Head*, slightly degraded image quality for *Thorax* and *Pelvis*	None	None
Mode	Coarse → Fine	Noise, uniformity, and contrast noticeably improved	Increased by up to a factor of 2	Increased by nearly a factor of 2

*Note*: It is assumed that for a change in each parameter, all other parameters remain identical. Listed modifications for each parameter include all other intermediate options (e.g., small, medium, large for body size).

Upon the analysis of the data, it is also necessary to discuss several possible limiting factors for ClearRT. First, the system uses an FPD purely because this detector is needed for planar Synchrony images and space limitations do not allow for the inclusion of a standard multi‐row CT detector. Generally, FPDs are reserved for CBCT systems (though *Coarse* mode for ClearRT is similar to a cone‐beam geometry) and have smaller scintillating crystals than standard CT detectors, resulting in lower signal intensity. Additionally, no anti‐scatter grid is used with the detector as physical scatter rejection is accomplished by narrowing the beam with the source side collimator. As a result, the lack of collimation at the detector results in greater scatter acceptance and possibly degraded image quality. Lastly, it is likely that ClearRT will be more susceptible to motion artifacts in practice compared to systems such as Siemens Edge due to a much slower gantry rotation. The impact of blurring on IGRT and ART implementation is yet to be seen, though the increased scan speeds of ClearRT compared to other IGRT imaging systems should alleviate some of this issue as patient breath holds throughout the duration of the scan are more likely.

Future work must be performed to assess the clinical impacts of these observations on the reliability of IGRT and ART processes. However, the analysis of this data does suggest that ClearRT provides sufficient image quality to delineate anatomic structures as required by IGRT and ART. Also, the maximum differences in CT numbers between protocols at identical energies for ClearRT compared to Siemens Edge does suggest that a limited number of calibration curves for dose computation may be required. A further evaluation of dose calculation accuracy is necessary to determine if this is indeed the case or if parameter‐specific calibration curves are needed at each energy.

ClearRT is intended for daily use in IGRT and/or ART implementation on the Radixact helical tomotherapy system, and the image quality improvement over the previously available MVCT system was evident in this study. Noise was reduced by approximately a factor of 2 for ClearRT and contrast metrics were improved by a factor of 3–4 when compared to Radixact MVCT with the *IR General* reconstruction algorithm, and these metrics are only further improved when compared to the *Standard* reconstruction algorithm on the MVCT systems. The “cupping” or “capping” artifact was also decreased significantly on average, and scan times for identical scan lengths were decreased by a factor of approximately 4–8 depending on acquisition parameters. Thus, it is evident that far better image quality can be achieved with the latest upgrade on the Radixact system when compared to what is currently available on most helical tomotherapy systems in practice. It is expected that this study can be used as a foundation by physicists to make responsible clinical decisions regarding useful imaging protocols for ClearRT.

## 5 CONCLUSION

Various image quality metrics have been evaluated for ClearRT and compared to those measured for systems established in IGRT and/or ART uses. ClearRT performed well in comparison for metrics most important to IGRT and ART applications for the delineation of anatomic structures—mainly noise and contrast. The results presented in this work provide a foundation for the optimization of this implementation in a clinical setting. An overall improvement in image quality is evident between ClearRT and the previously available MVCT system, and future work will be performed to continue to determine the clinical impact of the application of this new system.

## AUTHOR CONTRIBUTION

Riley C. Tegtmeier: conceptualization, data curation, formal analysis, investigation, methodology, writing—original draft.

William S. Ferris: conceptualization, investigation, methodology, writing—review and editing,

John E. Bayouth: conceptualization, supervision, writing—review and editing.

Jessica R. Miller: methodology, investigation, resources, writing—review and editing.

Wesley S. Culberson: conceptualization, methodology, resources, supervision, writing—review and editing.

## CONFLICT OF INTEREST

John E. Bayouth has ownership interest in MR Guidance, LLC, which has business activity with a company that also utilizes image‐guided radiation therapy technology (ViewRay, Inc.). Additionally, although this project received no external funding, the data was collected on a Radixact system (Accuracy, Inc.) provided to UW–Madison under a research agreement (Bayouth, PI) and Jessica R. Miller has research funding support from Siemens Medical Solutions.
